# Supplementary material for: Identification and characterization of CYPs induced in the Drosophila antenna by exposure to a plant odorant
Source: Sci Rep. 2021 Oct 15;11:20530. doi: 10.1038/s41598-021-99910-9 (PMC8521596; doi:10.1038/s41598-021-99910-9)
Supplement: Supplementary file 1 — Supplementary Figures. [file 41598_2021_99910_MOESM1_ESM.pdf]

Supplementary Information

Identification and characterization of CYPs induced in the *Drosophila* antenna by exposure to a plant odorant

Shane R. Baldwin, Pratyajit Mohapatra, Monica Nagalla, Rhea Sindvani, Desiree Amaya, Hope A. Dickson, and Karen Menuz

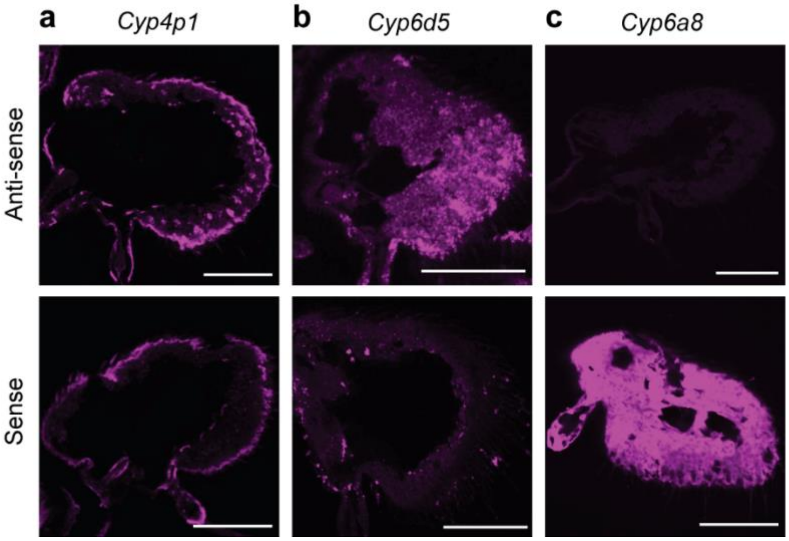

Supplementary Figure 1. FISH can be used to localize *Cyp4p1* and *Cyp6d5*, but not *Cyp6a8*. (**a**) Antennal sections from wild-type flies exposed to 5% geranyl acetate for two hours show cellular labeling with an anti-sense *Cyp4p1* FISH probe, but not a sense control probe. Non-specific cuticular labeling on the edge of the antenna is also observed. (**b**) Similar to (**a**), but for *Cyp6d5*. (**c**) A *Cyp6a8* anti-sense FISH probe does not label antennal sections from wild-type flies exposed to geranyl acetate, but non-specific staining is seen in sense probe controls. Scale bars are 50 µm in each image.

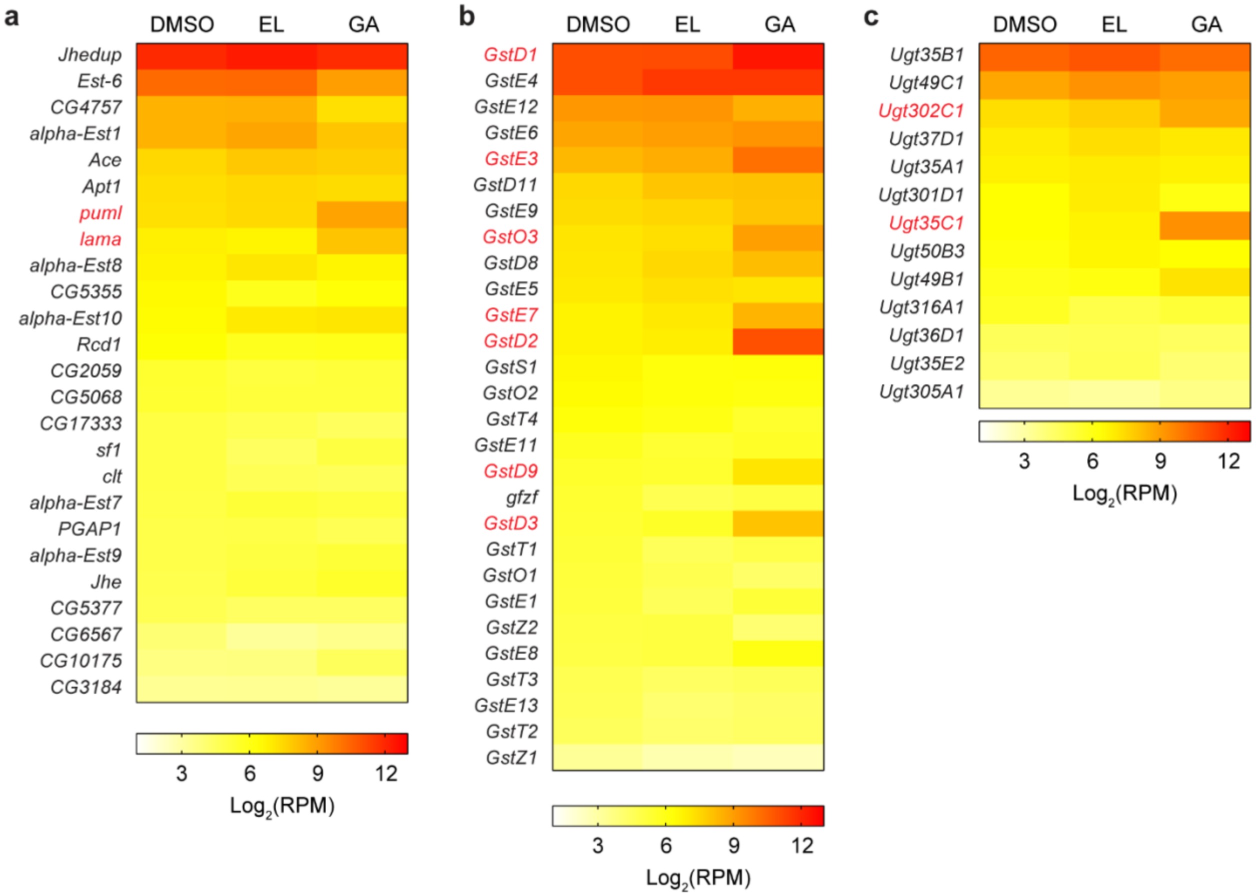

Supplementary Figure 2. Expression of other ODEs following odorant exposure. (**a**) A heat map showing the average expression in $\log_2$(RPM) of each of the antennal-expressed carboxylesterase genes in each odorant exposure condition (n=3). (**b**) Similar to (**a**), but for GSTs. (**c**) Similar to (**a**), but for UGTs. In each panel, GA-induced genes are in red font.
